# Mental disorders as networks of problems: a review of recent insights

**DOI:** 10.1007/s00127-016-1319-z

**Published:** 2016-12-05

**Authors:** Eiko I. Fried, Claudia D. van Borkulo, Angélique O. J. Cramer, Lynn Boschloo, Robert A. Schoevers, Denny Borsboom

**Affiliations:** 1 Department of Psychology, University of Amsterdam, Nieuwe Achtergracht 129-B, Room G0.28, 1001NK Amsterdam, Netherlands; 2 Department of Psychiatry, University Medical Center Groningen, University of Groningen, Groningen, The Netherlands

**Keywords:** Comorbidity, Early warning signals, Mental disorders, Network analysis, Treatment

## Abstract

**Purpose:**

The network perspective on psychopathology understands mental disorders as complex networks of interacting symptoms. Despite its recent debut, with conceptual foundations in 2008 and empirical foundations in 2010, the framework has received considerable attention and recognition in the last years.

**Methods:**

This paper provides a review of all empirical network studies published between 2010 and 2016 and discusses them according to three main themes: comorbidity, prediction, and clinical intervention.

**Results:**

Pertaining to comorbidity, the network approach provides a powerful new framework to explain why certain disorders may co-occur more often than others. For prediction, studies have consistently found that symptom networks of people with mental disorders show different characteristics than that of healthy individuals, and preliminary evidence suggests that networks of healthy people show early warning signals before shifting into disordered states. For intervention, centrality—a metric that measures how connected and clinically relevant a symptom is in a network—is the most commonly studied topic, and numerous studies have suggested that targeting the most central symptoms may offer novel therapeutic strategies.

**Conclusions:**

We sketch future directions for the network approach pertaining to both clinical and methodological research, and conclude that network analysis has yielded important insights and may provide an important inroad towards personalized medicine by investigating the network structures of individual patients.

**Electronic supplementary material:**

The online version of this article (doi:10.1007/s00127-016-1319-z) contains supplementary material, which is available to authorized users.

## Introduction

In the last years, a growing number of publications have studied mental disorders, such as Major Depressive Disorder (MDD), Post-Traumatic Stress Disorder (PTSD), and psychosis as networks of interacting symptoms. Although this scientific discipline is young, with its conceptual roots in 2008 [1] and its empirical foundations in 2010 [2], it is fast-moving and has gained considerable recognition. The big step forward within the last years has been the development of statistical models that allow for the estimation of empirical psychopathology networks. The present paper aims to provide a review of the contemporary empirical literature on this network conceptualization of psychopathology. For more information on the methodology behind these empirical papers—network psychometrics—we refer the interested reader elsewhere [3–9].

**Fig. 1:**
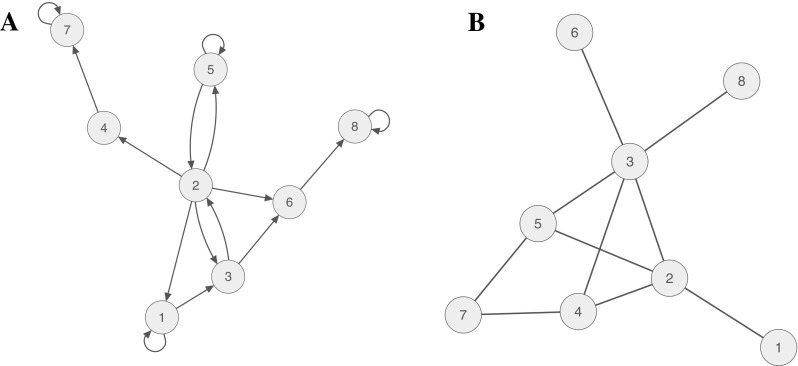
**a** Pairwise associations among eight symptoms of a hypothetical patient Susan; this network is based on time-series data and thus leads to a directed network. **b** Pairwise associations among eight symptoms in a hypothetical group of patients; this network is based on cross-sectional data and thus leads to an undirected network

According to the network perspective on psychopathology, a mental disorder can be viewed as a system of interacting symptoms. From this perspective, the causal interplay between symptoms constitutes mental disorders [2, 10, 11]. Taking Major Depressive Disorder (MDD) as an example, depressed patients often experience symptoms, such as sadness, anhedonia, fatigue, insomnia, concentration problems, and suicidal ideation [12], and it is easy to envision causal relationships among these problems, for instance, fatigue → insomnia → concentration problems, or sadness → anhedonia → suicidal ideation. Figure 1a shows an example of such a directed network for a hypothetical depressed patient Susan. Figure 1b, on the other hand, depicts an undirected network estimated in a group of people in cross-sectional data (the syntax to reproduce all figures is available in the Supplementary Materials).

The remainder of the paper is organized into four sections. First, we review publications that aim to explain the comorbidity rates among mental disorders using the network approach. Second, we summarize network studies that have been conducted with the aim of predicting the course of disorders, and to identify indicators of a worse prognosis. Third, we discuss what insights network studies have yielded for clinical intervention. Finally, we discuss implications for future clinical practice and how the network perspective can move forward.

## Comorbidity

The presence of multiple disorders at the same time is extremely common in the realm of psychopathology [13]. Comorbidity has received considerable attention in the clinical literature, because patients diagnosed with multiple disorders have poorer prognosis, worse treatment outcomes, and higher suicide rates [14, 15].

### Comorbidity from a network perspective

**Fig. 2:**
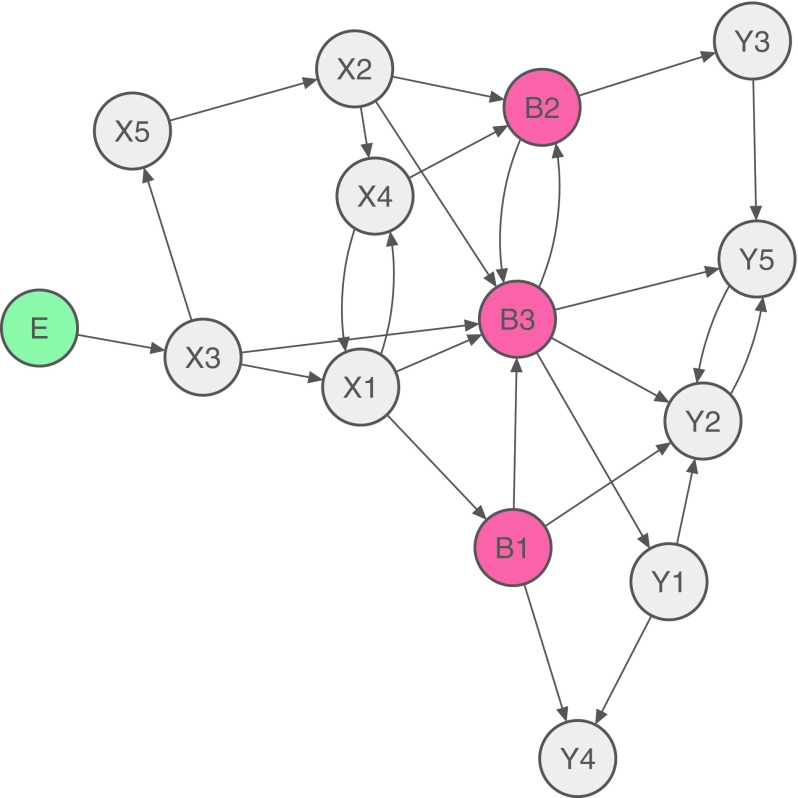
How comorbidity can arise according to the network approach. Disorder X consists of the eight symptoms X1–X5 and B1–B3, and disorder Y consists of the eight symptoms Y1–Y5 and B1–B3. B1–B3 are bridge symptoms that feature in both diagnoses. In this case, a person first develops X3 in response to an environmental stressor E, then symptoms of disorder X, then bridge symptoms B, and finally symptoms of disorder Y

Traditionally, comorbid mental disorders are understood as different disorders, while the network approach hypothesizes that they may co-occur due to mutual interactions among symptoms [2]. Comorbidity, in that view, arises when there are symptoms that bridge two disorders. These so-called bridge symptoms can spread activation from one disorder to the other. Figure 2 represents such a case where a person first develops disorder X (in response to an environmental stressor E), then the bridge symptoms B, and finally disorder Y. X could be MDD, Y could be Generalized Anxiety Disorder (GAD) that often occurs together with MDD, and the bridge symptoms B could be sleep problems, fatigue, concentration problems, or psychomotor agitation that are part of both MDD and GAD DSM-5 criteria [12].

Note that there are numerous other possibilities from a network perspective to explain the comorbidity between X and Y: activation may go the other way around (Y → B → X), or a person could also develop the bridge symptoms B first, and then at the same time both X and Y.

### Comorbidity in empirical data

Several cross-sectional studies have investigated how symptoms are related across disorders. In the first empirical network study on the subject, Cramer et al. [2] found that the empirical network structure of MDD and GAD symptoms in a general population sample was entangled. A recent paper replicated these findings in a large clinical sample, also concluding that MDD and GAD symptoms are strongly interconnected [16]. Another team of researchers studied the comorbidity of MDD and complicated grief [17], and showed that symptoms form two distinct clusters that are connected through the symptoms loneliness, emotional pain, and emotional numbing. The authors suggested that emotional pain may be a promising target for psychotherapeutic interventions.

Boschloo et al. [18] used a network analysis in a data set with over 34,000 patients interviewed on 120 symptoms of 12 major DSM-IV disorders. In the resulting network, no sharp boundaries were found between the 12 disorders (i.e., symptoms of different diagnoses were related to each other across diagnoses), and there was substantial symptom overlap across diagnoses. The authors repeated this analysis in a community sample of 2175 preadolescents with 95 emotional and behavioral problems, with similar results [19].

Comorbidity research from a network perspective has also generated new hypotheses for Autism Spectrum Disorder (ASD) and Obsessive–Compulsive Disorder (OCD), both of which share repetitive behaviors. A study in a clinical sample of 213 children revealed that repetitive behaviors seem to connect ASD and OCD symptom clusters, and the authors also found evidence that repetitive behaviors may differ somewhat in people with ASD and people with OCD [20].

One implication of the network view on comorbidity is that diagnoses may co-occur as a function of their number of shared symptoms. As described above, MDD and GAD should have relatively high comorbidities, whereas disorders that share no symptoms, such as schizophrenia and specific phobias, should rarely co-occur because of the lack of bridge symptoms that can transport information. Interestingly, this implication does not derive from the traditional conceptualization of mental disorders as medical conditions, where disorders cause their symptoms [21, 22]. The fact that diagnoses for HIV, cancer, and tuberculosis may share more or less symptoms should not impact strongly on their rates of comorbidity, seeing that they have independent common causes. One study tested that prediction and measured how related the networks of different mental disorders are. They found that empirical comorbidity rates were related to distances between disorders in an analysis of symptom overlap in the DSM [23].^1^ This means that when two disorders, such as MDD and Dysthymia, share multiple symptoms, the distance between these disorders in the DSM network is small and one can easily travel from one disorder to the other.^2^

To conclude, zooming in on disorders at symptom level [25, 26], as the network perspective does, reveals how comorbidity might come about. Currently, this issue is empirically unresolved, and the way comorbidity arises may very well be different for different people with the same comorbid diagnoses and different for different types of comorbid diagnoses.

## Prediction

While many people experience single symptoms, only part of them develop a mental disorder. One of the most important areas of clinical research is thus the prediction of psychopathology onset, which would allow clinicians earlier interventions. The network literature on prediction has, thus far, focused on two aspects: (1) so-called early warning signals that may indicate the upcoming onset of psychopathology for a specific patient and (2) characteristics of group-level networks that may help predict the future course of psychopathology. It is of note that the work on prediction has mostly investigated emotion dynamics [27]—the temporal associations between emotions, such as sadness, anger, fear, or being content—while little research has been conducted on the dynamics among a broader set of problems or symptoms like insomnia, fatigue, and concentration problems. Such investigations are a topic of future research [28].

### Early warning signals

The conceptualization of mental disorders as networks of interacting symptoms allows utilizing insights from scientific fields in which complex systems are well known. One of the most important features of complex systems in this regard is that they can display phase transitions [29] which mark the transitions between healthy and ‘disordered’ states [30]. Identifying early warnings of such transitions is a promising line of research known from other fields, such as ecology or financial markets, where systems can reach a tipping point [29–31]. Lakes, for example, can shift from a clear state to a turbid state, and it is an important question how to best predict such tipping points. Interestingly, right before such phase transitions from one state to another, a system displays early warning signals. Specifically, transitions are preceded by a phenomenon referred to as critical slowing down [29, 32, 33], which means that it takes longer for a system to recover from perturbations. This is reflected by the fact that the system becomes more predictable by its previous states: when close to a transition, the dynamics slow down.

We can use the network of Susan (Fig. 1a) as an example of a bi-stable system with two attractor states: a healthy and a sick state. Assume Susan is now in a healthy state, and an early warning signal would indicate an upcoming tipping point, where her system may suddenly move from healthy to sick. Before a transition occurs, the system slows down, which implies that we can more reliably predict the state of the system at the next time point. In statistical terms, one such sign is increasing autoregressive coefficients (i.e., the self-predictive pathways from a symptom or emotion to itself across time).

Van de Leemput et al. [30] estimated emotion dynamics during critical transitions from healthy to depressed states to see whether these are preceded by early warning signals. Analyzing a large time-series data set, the authors showed that systems exhibited signs of critical slowing down before critical transitions [30]. A second study on early warning signals was published recently on one depressed patient that was measured over 239 days (1474 measurements). This intensive idiographic study, in which the patient decreased his antidepressant intake, also shows evidence for early warning signals before he transitioned into a depressive episode [34]. There is also some work showing that individuals with higher levels of inertia in their emotion dynamics are more likely to develop depression 2.5 years later [35]. Inertia also refers to auto-correlations and implies that emotion networks of individuals at higher risk to develop depression on the long term are characterized by a slower recovery from a given perturbation (i.e., the emotion networks of people at higher risk might recover more slowly from the effect of external influences than those at lower risk). While further investigations are required to understand the nature of emotional inertia, it has been considered “a hallmark of maladaptive emotion dynamics” [27] (p. 984).

An interesting topic in this context is the observation that phase transitions may be more pronounced with increasing levels of connectivity. That is, for weakly connected symptom networks, negative external conditions (i.e., stressful events) lead to a gradual increase in symptoms, whereas for strongly connected networks, external stress leads to a sudden shift from a healthy to depressed state [30]; simulation studies with depression networks support this notion [28, 36]. This may shed new light on a long-standing discussion whether psychopathology is dimensional or categorical [37]: networks with weak connectivity may behave as a continuum in response to stress (i.e., no sudden phase transition; psychopathology is dimensional), while networks with strong connectivity may behave as either healthy or disordered (i.e., sudden phase transitions; psychopathology is categorical). This implies that different people may have the same diagnosis, for instance MDD, but that the connectivity of the network structure would determine whether the disorder is a continuum or a dimension for them.

### Prediction via network characteristics

Another important aspect of network research is the prediction of the course of psychopathology from network characteristics of groups of individuals. Two studies showed that the temporal emotion networks of patients with MDD and psychosis [38, 39] were more strongly connected than the temporal emotion networks of healthy controls. Stronger temporal connections between emotions mean that the state of an emotion at a certain timepoint depends strongly on the state of emotions at the previous timepoint. In another study, van Borkulo et al. [40] hypothesized that higher levels of connectivity in depressed patients at baseline are associated with worse outcomes at 2-year follow-up. They found that patients with persistent depression at follow-up had a more densely connected cross-sectional network at baseline compared to remitted patients at follow-up—even after controlling for differences in severity [40]. This is consistent with other work arguing that more densely connected temporal network structures may be more vulnerable to psychopathology [41]. Whether this also applies to particular individuals (in contrast to network structures at the group level), however, remains to be investigated with within-person analyses [42, 43].

Another study on predicting the future course of psychopathology showed that the most interconnected or central depression symptoms in the baseline network were the ones most predictive of future MDD onset (Boschloo et al. [44]). Fatigue and depressed mood, for example, were more predictive of MDD than other symptoms. This implies that the nature of symptoms may play an important role above and beyond the number of symptoms [45].

## Clinical intervention

Network analysis may provide promising leads towards improving clinical prevention and intervention strategies by investigating which symptoms are more strongly connected or more central than others. In this section, we will first explain the concept of centrality, and then discuss the results of research that points to possible targets for intervention.

### The concept of centrality

**Fig. 3:**
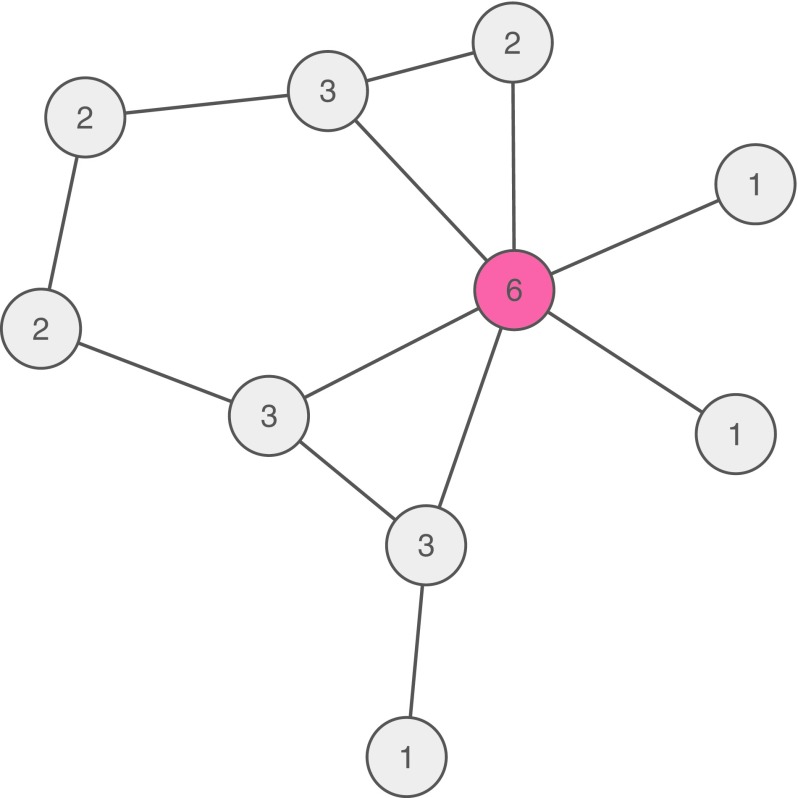
Psychopathological network showing the pairwise associations among ten symptoms. Each node depicts a number that is the sum of all connections of the node with all other nodes, called degree centrality. The red node has the highest degree centrality with six connections

If a symptom (e.g., depressed mood) has many connections to other symptoms in a psychopathological system, it may cause the development of these symptoms. The number of connections of a symptom is known as degree centrality. This type of centrality is illustrated in Fig. 3: the red symptom is connected to six other symptoms, whereas all other nodes have a lower number of connections (as indicated by the numbers in each node). The red node thus has a high (degree) centrality and, consequently, may be seen as a risk factor for developing further symptoms [4, 10, 46–48].

That is, if someone develops a symptom that is central, the probability of developing other symptoms will increase more than when someone develops a peripheral symptom.

Degree centrality can be understood to quantify the importance of a node in the network. Other common centrality measures are closeness and betweenness [24], and several papers have described and calculated these centrality measures for psychopathological networks [46, 48]. In directed networks (e.g., longitudinal network models, where nodes predict other nodes over time), degree can be further specified with indegree being the number of connections pointing towards the focal symptom and outdegree being the number of connections pointing from the focal symptom to other symptoms (cf. [4]. Especially, symptoms with a high outdegree might be viable targets for intervention, since they influence many other symptoms.

### What are good symptoms for clinical intervention?

Providing an overview of which symptoms are more central than others across studies turns out to be a challenging task for several reasons. Different studies used different variables, making comparisons across studies challenging. For MDD, for instance, different questionnaires were used to assess symptomatology, or researchers analyzed other types of variables, such as emotions or mental states [38, 39, 49]. Other factors that vary across studies are the temporal nature of the data (cross-sectional vs. time-series data), the particular samples studied (e.g., healthy, moderately depressed, and severely depressed samples), and the network estimation methods (e.g., [4, 50]). With these caveats in mind, it seems that the two DSM-5 [12] core symptoms of MDD episode—depressed mood and loss of interest/pleasure—along with energy/fatigue consistently appear as central symptoms and could thus be understood as potential targets for intervention [2, 19, 46, 51, 52].

Since other disorders have not been investigated as frequently, it is not possible to identify similarities across studies. Therefore, we summarize the available literature, urging researchers to replicate these results before translating them into clinical settings. A cross-sectional network study of 2405 adults with different substance abuse disorders revealed that using the substance longer than planned and that the drug interferes with life significantly were highly central [53]. Interestingly, these problems appear in the later course of the disorder, and provide a great example for central symptoms that may not prove to be the best targets for clinical intervention. The symptoms may be central in the cross-sectional substance abuse study primarily because they often develop as a consequence of other symptoms. Rhemtulla et al. also found that the most central symptoms in the full sample differed considerably in their centrality across subgroups of people with different types of substance use disorders (cannabis, sedatives, stimulants, cocaine, opioids, and hallucinogens). For PTSD, McNally et al. concluded from analyzing cross-sectional data that hypervigilance, impaired concentration, and physiological reactivity to reminders of the trauma are promising targets for intervention [54]. Sleep difficulty was also among the most central symptoms, and aiming to stabilize patients’ sleep might be a promising strategy (even before initiating other forms of psychotherapy), which could induce a cascade of symptom deactivation.

Researchers have also included variables other than the symptoms of the disorder itself in networks. First, a cross-sectional study on psychosis included information on childhood trauma in the network of psychosis and psychopathology symptoms, such as anxiety and depression. Different types of childhood trauma were related to psychosis symptoms, but only through general psychopathology symptoms, such as anxiety [55]. Second, protective (resilience) variables might also be included in psychopathological networks (for a brief discussion, see [23]). To our knowledge, only one cross-sectional study investigated this, though not on a symptom level [56]. The authors found resilience to be related to remission of depression and showed it was central in a network with composite scores of other cognitive processes, such as cognitive control, experienced cognitive functioning, maladaptive emotion regulation, and residual depressive symptomatology. Third, Heeren and McNally [57] investigated the cross-sectional network structure of the core symptoms (fear and avoidance) of Social Anxiety Disorder (SAD) with laboratory measures on attention bias. They found that attention bias played an important role in the network, arguing that process-level measures from laboratory tasks can shed more light on the mechanisms of SAD (Heeren and McNally [57].

## Future directions

Much exploratory network research has been conducted in the field of psychopathology network research: but where do we go from here? In this section, we will discuss some future perspectives, structured into clinical and methodological research.

### Clinical research

From a clinical perspective, we suggest to investigate four topics. First, the network framework generates specific hypotheses about treating disorders that should be explored. In treating comorbid disorders, such as MDD and GAD, for example, targeting bridge symptoms that transfer influence from one part of the network to the other should be the strategy of choice. A related hypothesis is that targeting central symptoms should reduce patients’ symptomatology [46].^3^ As the majority of research on finding possible targets for intervention is based on cross-sectional data, it is unclear whether an undirected edge between symptoms A and B implies A → B, A ← B, or A ⟷ B.^4^ Longitudinal analyses allow for an estimation of directed networks which reveal the direction of the association between symptoms, such as A and B (e.g., [51]), and present a more promising route to investigate possible targets for clinical intervention.

Second, and related to the previous point, it should be investigated whether intervening on central symptoms will actually bring benefits to patients. Although studies have collected ESM data in therapeutic settings to provide feedback on patterns of affect [58–60], these data have not been analyzed using network models to derive, for instance, the most central symptoms—and a large crowdsourcing study that does provide feedback via personalized dynamic networks does so only outside a therapeutic setting [61]. Merging these two approaches may provide valuable insights, and we are aware of one such pioneering case study that investigated personalized feedback based on network models within a therapeutic setting [62]. In addition to treatment as usual, the patient received feedback on symptom dynamics and explored the feasibility, acceptability, and usability of such an integrated individualized network approach. This initiated a therapeutic dialogue about possible causes of treatment resistance and may provide new directions towards personalized medicine [63, 64]. While it may not always be feasible or possible to target a specific symptom, establishing that the network framework provides good explanatory and predictive models of psychopathology may imply the need for developing new approaches for targeting specific symptoms.

Third, it would be worthwhile to apply the network perspective to yet unexplored mental disorders. The temporal dynamics of symptoms of Binge Eating Disorder (BED), for example, may be a suitable candidate [12]. One causal pathway could be between the symptoms eating until one feels uncomfortably full and feeling disgusted, depressed, or guilty, which could provide insights into risk factors for the development of BED episodes.

Fourth, besides looking at the interactions of problems (e.g., symptoms), studying factors that contribute to resilience may be worthwhile pursuing [65, 66]. Investigating the role of protective factors in psychopathology networks might inform us how these two opposing forces relate to each other, and eventually inform clinical practice. For example, Alice may benefit from more social interactions in case social isolation leads to sad mood, while Bob may benefit from physical activity in case sad mood is preceded by lack of activity.

### Methodological research

Exploring the above questions relies on the accurate and reliable *estimation* of psychopathological networks. When patients apply for treatment,^5^ there is often a waiting period in which one could assess the emotion and symptom dynamics with modern phone technology within an idiographic momentary assessment study, and results could inform treatment. Similarly, relapse prevention in remitted patients may benefit from repeated assessment of core symptoms and related factors over time to foresee relapse in an early phase and take preventive measures to counteract its course. This all sounds promising, but before this can be put into effect, there are some methodological issues that need to be addressed of which we will discuss three.

A first issue is what variables to study in psychopathological networks. While cross-sectional network studies have focused on analyzing associations among symptoms, ESM studies have focused on mood states, such as sadness, happiness, anxiety, or anger [4, 38, 49, 67]. It is unclear at present what level of variables is best to study psychopathology.

A second issue is the time frame on which to measure symptoms or emotions. In most ESM studies, the time frame between measurements is a few hours. However, do symptoms or affects change within hours or minutes or days? This might differ for different pairs of symptoms: experiencing somatic arousal (e.g., increased heart rate and sweating) might lead to anticipating a panic attack [43], which will occur within minutes. Sleep problems, on the other hand, might build up for a few days before influencing a person’s irritability. It is currently unknown what the best timeframe is to capture dynamics.

Third, an important point is the generalization of group-level results to the individual level, since many group-level network studies have implied that the identified network structure of the population is more or less reflective of the networks of all individual participants (e.g., [68, 69]). A well-known example of this phenomenon, known as Simpson’s Paradox, is the speed–accuracy tradeoff. At a group-level, a negative relationship exists between typing speed and typing accuracy: people with higher typing speed make fewer errors, likely because experience leads to faster typing and fewer mistakes. At the individual level, however, a person who types faster will make more, not less errors [70]. While this is an extreme example—it seems unlikely that symptoms of mental disorders are predominantly positively associated at group-level, but negatively in the individual—we currently do not know to what extent group-level networks differ from individual networks [43]. A related point was made by Bos and Jonge [71] and Bos and Wanders [42] who warn that between-person effects should not be confused with within-person effects. Taken together, this implies that we need future studies that investigate to which degree idiographic networks match group-level networks, and to disentangle between-person from within-person effects.

Finally, numerous network papers analyzed data that contained a skip structure. This is often the case when large populations are screened via the DSM diagnostic criteria. For a diagnosis of MDD, for instance, subjects need to endorse at least one of the two core symptoms depressed mood or anhedonia. If that is not the case, the remaining seven MDD symptoms are skipped. In statistical analyses, such skipped items are usually recoded as 0s (e.g., [10, 19, 53]), but just because someone does not endorse the core symptoms does not mean that the person cannot exhibit other MDD symptoms. The recoding of missing data to 0s may pose a considerable problem, because it introduces spurious correlations among items (for many people, the seven remaining items will be coded as 0s and thus be highly correlated, although this may not reflect the true correlations among items). Although Boschloo et al. [18] showed similarity of the network structure based on the original data with 49% missing and a subsample with less than 20% missing, it still may have introduced bias. Future research is required to investigate imputation strategies for skip data that go beyond recoding them as 0s.

## Summary

In contrast to current categorical diagnostic classifications that hardly fit clinical reality, the network approach offers a model that captures both complexity and individual variation in psychopathology that clinicians and patients immediately recognize. Due to recent statistical advances, these networks and the resulting hypotheses can now be empirically tested and validated, both in nomothetic and idiographic (*n* = 1) designs. Electronic devices, such as smartphones, watches, and other ‘wearable tech’ offer the possibility of continuous/repeated data collection to address important clinical issues regarding vulnerability for and onset of psychopathology as well as relapse prevention. However, it is also likely to be helpful in regular therapy as it enhances patients’ insight into their own symptom dynamics and how these relate to contextual and behavioral factors that they themselves may be able to influence. Pilot studies suggest that this type of objective and differentiated feedback attributes to traditional ‘talking therapy’ and may also lead to more informed pharmacotherapy [72]. Taken together, the network approach offers a promising conceptual framework to further develop personalized medicine in psychiatry.

## Electronic supplementary material

Below is the link to the electronic supplementary material.

Supplementary material 1 (TXT 5 kb)

## Footnotes

1 Note that this DSM network is not based on empirical data, but each node in the network is a symptom as listed in the DSM. Overlapping symptoms are depicted as one and the same node.

2 The network *distance* between symptoms of different disorders is based on the *average shortest path length*. This measure is calculated by averaging the number of steps (i.e., the number of connections) to travel from each symptom of one disorder to each symptom of the other disorder [24].

3 The simulation tool "vax" provides a beautiful explanation of centrality and shows how such treatment should work by targeting the most central nodes: http://vax.herokuapp.com/game.

4 Note that there are two further possibilities that can explain an undirected edge between A and B: an unmodeled influence of a (set of) latent variables C (A ← C → B); and conditioning on a common effect D such that A → D ← B. The latter can arise if D is a variable included in the network, but also when it is not, for instance when the investigated sample has been selected on (a function of) a common effect of the variables in the network.

5 For an ongoing study on this topic, see http://www.trialregister.nl/trialreg/admin/rctview.asp?TC=5707.
